# Classical and Quantum H-Theorem Revisited: Variational Entropy and Relaxation Processes

**DOI:** 10.3390/e23030366

**Published:** 2021-03-19

**Authors:** Carlos Medel-Portugal, Juan Manuel Solano-Altamirano, José Luis E. Carrillo-Estrada

**Affiliations:** 1 Instituto de Física, Benemérita Universidad Autónoma de Puebla, Apdo. Postal. J-48, Puebla 72570, Mexico; cmedel@ifuap.buap.mx; 2 Facultad de Ciencias Químicas, Benemérita Universidad Autónoma de Puebla, 14 Sur y Av. San Claudio, Col. San Manuel, Puebla 72520, Mexico; jmanuel.solano@correo.buap.mx

**Keywords:** non-equilibrium thermodynamics, entropy, variational entropy

## Abstract

We propose a novel framework to describe the time-evolution of dilute classical and quantum gases, initially out of equilibrium and with spatial inhomogeneities, towards equilibrium. Briefly, we divide the system into small cells and consider the local equilibrium hypothesis. We subsequently define a global functional that is the sum of cell *H*-functionals. Each cell functional recovers the corresponding Maxwell–Boltzmann, Fermi–Dirac, or Bose–Einstein distribution function, depending on the classical or quantum nature of the gas. The time-evolution of the system is described by the relationship dH/dt≤0, and the equality condition occurs if the system is in the equilibrium state. Via the variational method, proof of the previous relationship, which might be an extension of the *H*-theorem for inhomogeneous systems, is presented for both classical and quantum gases. Furthermore, the *H*-functionals are in agreement with the correspondence principle. We discuss how the *H*-functionals can be identified with the system’s entropy and analyze the relaxation processes of out-of-equilibrium systems.

## 1. Introduction

The theoretical bases and the procedures that allow us to describe equilibrium systems are well-established. These procedures can be applied to a wide range of natural systems, including both the macroscopic phenomenological methods (thermodynamics) and the microscopic description (statistical mechanics) (Out-of-equilibrium systems, of course, are still a challenge). For instance, in the kinetic theory of gases, the behavior of a dilute classical gas is described through the Boltzmann transport equation [1], and the time-evolution of a system towards equilibrium is finely accounted for through the Boltzmann *H*-theorem.

However, for quantum out-of-equilibrium systems, the construction of a kinetic framework with the same level of success and universality as the classical version still presents some fundamental challenges. For instance, to obtain a complete correspondence principle between classical mechanics and quantum mechanics, the form of the quantum analogues of both the Boltzmann *H*-theorem and the Boltzmann transport equation is inadequate. In this context, Tolman was one of the earliest physicists to propose a quantum *H*-theorem [2], using a probability transition relationship, the random phases hypothesis, and an *H*-functional defined in terms of a spatially homogeneous distribution function. Tolman also proposed a potential quantum analogue of the transport equation, in terms of the occupation numbers, by applying time perturbation theory. Additional attempts, under quantum operator formalism, have addressed the description of quantum transport phenomena through the Hamiltonian of the system and the master equation (which is, in these works, the analogue of the Boltzmann transport equation) [3,4,5,6,7]. However, these approaches are not consistent with the classical-quantum correspondence principle. Similarly, some authors have proposed *H*-functionals and attempted to proof a quantum *H*-theorem [8,9,10,11,12,13,14,15,16]. However, whether or not the homogeneous distribution function hypothesis is assumed or if its framework fulfills the correspondence principle is unclear or not discussed. Since the pioneering work of Tolman, at several stages, there has been some discussion regarding the general validity of the quantum *H*-theorem, some possible violations of the second law of thermodynamics, and the interpretation of the quantum entropy [10,12,13,14,16,17,18,19,20,21].

Nonetheless, the framework to describe spatially non-homogeneous systems is still under construction, although several approaches have been developed. For instance, the celebrated Onsager formulation (linear thermodynamics) [22,23] has been successful in describing irreversible chemical and physical phenomena. However, some descriptions, such as those the internal behavior of gases [24] and the entropy measurement [25,26], cannot be completely addressed with linear thermodynamics.

In addition, some aspects regarding the classical *H*-theorem and the Boltzmann *H*-functional require revision to improve their mutual consistency. One example is the modification of the *H*-theorem to include phenomena stemming from stochastic trajectories, violations of the second law of thermodynamics, the relationship between Shannon’s measure of information and the Boltzmann’s entropy, and the calculation of thermodynamical quantities and thermalization of specific systems [8,26,27,28,29].

To contribute to the construction of a consistent classical and quantum *H*-theorem, within a formalism that describes out-of-equilibrium non-homogeneous systems, we propose a new theoretical framework. Specifically, for both classical and quantum systems, we include non-homogeneous distribution functions in the *H*-functionals, and consider non-homogeneous systems in the proofs of the resulting *H*-theorems. Our proposed *H*-functionals satisfy the correspondence principle, but more importantly, these functionals describe the time-evolution of spatially non-homogeneous systems towards equilibrium.

The organization of this article is as follows. In Section 2, we highlight, for our purposes, the most fundamental assumptions required to proof the Boltzmann *H*-theorem, we provide an alternative method to obtain the Maxwell–Boltzmann distribution using the variational method, propose an alternative *H*-functional for classical systems, and demonstrate the respective *H*-theorem. In Section 3, we review the Tolman proposal for the quantum version of the *H*-theorem (quantum *H*-theorem) and how the Bose–Einstein and Fermi–Dirac distributions are treated within this framework. Subsequently, we present our proposal for a quantum *H*-functional and the proof of the corresponding quantum *H*-theorem. In Section 4, we analyze the classical-quantum correspondence between the quantum and classical *H*-functionals. In Section 5, we explore how relaxation processes occur in a quantum ideal gas and, based on what we call variational entropy, propose a time-evolution equation for the distribution function. Finally, we discuss some key ideas resulting from our approach and close with a summary in Section 6.

## 2. Classical Scheme

The Boltzmann kinetic theory of gases represents a fundamental connection between the microscopic nature of matter and the phenomenological macroscopic laws of classical thermodynamics. The stochasticity introduced by the molecular chaos hypothesis in the otherwise deterministic kinetics of the particles allows for the demonstration of the celebrated Boltzmann *H*-theorem. In contrast, in this article, we propose an alternative approach developed using a variational procedure applied to an *H*-functional. We start this section by briefly accounting for the important elements of the standard derivation of the Boltzmann transport equation and demonstrating the *H*-theorem, such as they are presented in classical textbooks [1].

### 2.1. The Boltzmann Transport Equation

The first step in the Boltzmann kinetic theory of gases is defining the distribution function, $f(\vec{r},\vec{v},t)$, as the average number of molecules that, at time *t*, have position $\vec{r}$ and velocity $\vec{v}$, and are contained in a μ-space volume element $d^{3}rd^{3}v$. Assuming a deterministic Newtonian description of molecular motion, as well as the invariance of the μ-space volume measure, one arrives at the Boltzmann rate equation:

(1) $$f\left(\vec{r}+\vec{v}\delta t,\vec{v}+\vec{F}\delta t,t+\delta t\right)=f\left(\vec{r},\vec{v},t\right)+{\left(\frac{\partial f}{\partial t}\right)}_{\mathrm{coll}}\delta t.$$

Here, the term ${\left(\partial f/\partial t\right)}_{\mathrm{coll}}$ describes the in and out fluxes from and towards the volume element, due to the collisions. Subsequently, from the previous equation, the integro-differential Boltzmann transport equation is obtained:

(2) $$\left(\frac{\partial }{\partial t}+{\vec{v}}_{1}\cdot \nabla_{\vec{r}}+\frac{\vec{F}}{m}\cdot \nabla_{{\vec{v}}_{1}}\right)f_{1}=\int d\Omega \int d^{3}v_{2}\sigma \left(\Omega \right)\left|{\vec{v}}_{1}-{\vec{v}}_{2}\right|\left({\tilde{f}}_{2}{\tilde{f}}_{1}-f_{2}f_{1}\right).$$

In Equation (2), Ω is the solid angle, σ is the scattering cross section, $\vec{F}$ the external force applied to the system, and $f_{1}$ and $f_{2}$ (${\tilde{f}}_{1}$ and ${\tilde{f}}_{2}$) are the distribution functions of particles 1 and 2, respectively, before (and after) the collision.

Particle dynamics and the effects of external forces are described by the left-hand side of Equation (2). The right-hand side is derived by considering binary collisions between particles and accepting the *molecular chaos hypothesis*, i.e., it is assumed that the positions and velocities of the particles are not time-correlated.

### 2.2. A Summary of the H-Theorem and the Maxwell—Boltzmann Distribution

The evolution of a dilute gas towards thermodynamic equilibrium is frequently addressed by first defining the *H*-functional [1,2]:

(3) $$H_{B}=\int f\left(\vec{v},t\right)\ln f\left(\vec{v},t\right)d^{3}v.$$

Notice that $f_{B}\left(\vec{v},t\right)$ is a spatially homogeneous distribution function. The functional $H_{B}$, originally introduced by Boltzmann in 1872, describes a dilute gas occupying a volume *V*, at temperature *T*, with total energy *E*, and total number of free classical particles *N*. To clearly distinguish the Boltzmann functional $H_{B}$, we denote hereafter the Maxwell–Boltzmann distribution function as $f_{B}$.

The physically correct spontaneous time-evolution of an out of equilibrium dilute gas is corroborated by the *H*-theorem. This theorem establishes that if (a) the homogeneous function $f(\vec{v},t)$ satisfies the Boltzmann transport equation and (b) the molecular chaos hypothesis is valid, then the system evolves in such a manner that $dH_{B}/dt\leq 0$, and if $dH_{B}/dt=0$, then the system is in the equilibrium state. The *H*-theorem is straightforward to prove using Equation (2) [1], and it assures the consistency between our microscopic approach to describe the system’s spontaneous time-evolution and the phenomenological observations established by the second law of classical thermodynamics; in fact, $H_{B}$ can be associated with an entropy density.

On the other hand, considering a dilute gas in equilibrium with no applied external forces, i.e., (∂f/∂t)=0 and *f* is independent of $\vec{r}$, we can directly prove that the equilibrium distribution function obtained from Equation (2) is precisely the Maxwell–Boltzmann distribution function. The proof of the above first requires identification of the sufficient condition for *f* to render a null r.h.s. of Equation (2). Such an *f*, which we denote here as $f_{0}$, must satisfy

(4) $$f_{0}\left({\vec{v}}_{2}^{\prime }\right)f_{0}\left({\vec{v}}_{1}^{\prime }\right)-f_{0}\left({\vec{v}}_{2}\right)f_{0}\left({\vec{v}}_{1}\right)=0.$$

Subsequently, the Maxwell–Boltzmann distribution function can be obtained by taking the logarithm of Equation (4) and conserved mechanical quantities (see ([1], ch. 4.2)).

Before introducing our proposed *H*-functional, we must state that in defining $H_{B}$, Equation (3), it is assumed that the distribution function *f* is spatially homogeneous. This assumption simplifies the demonstration of the *H* theorem. However, it also introduces a limited conception of the out-of-equilibrium condition of the gas. Given the relatively simple nature of a dilute gas, one of the salient features of an out-of-equilibrium condition is the existence of inhomogeneities in the system, which is not considered in the above.

### 2.3. Non-Homogeneous Classical *H*-Functional

As we saw in the previous section, the validity of the *H*-theorem relies significantly on assuming that the distribution function is homogeneous and the molecular chaos hypothesis is fulfilled. To extend the previous procedure to systems with non-homogeneous distribution functions, which might allow for the study of systems in a more general out-of-equilibrium condition, we introduce a modified *H*-functional. For the sake of clarity and simplicity, we use primed functions and quantities to denote the classical case to differentiate them from the quantum analogues.

Our proposed classical *H*-functional, denoted as H′, describes a dilute classical gas occupying a volume *V*. In our theoretical treatment, we divide this volume into *K* cells, which, without loss of generality, have identical volumes, $\delta V_{M}=V/K,\;M=1,\;\cdots \;,K$. Each cell of index *M* has the following local functions, properties, and variables: An *H*-functional, $H_{M}^{\prime }$, a homogeneous distribution function, $f_{M}^{\prime }\left(\vec{v},t\right)$, number of particles, $N_{M}^{\prime }$, temperature, $T_{M}^{\prime }$, and energy, $E_{M}^{\prime }$. Taken as a whole, the system has an energy *E*, and a global number of free classical particles *N*. We also assume that the system is perfectly isolated, and that the number of particles in each cell is sufficiently large, so as to obtain accurate averages. We start our analysis by proposing the following inhomogeneous *H*-functional:

(5) $$H^{\prime }\left(t\right)=\sum_{M=1}^{K}\int_{\delta V_{M}}f_{M}^{\prime }\left(\vec{v},t\right)\ln f_{M}^{\prime }\left(\vec{v},t\right)d^{3}v.$$

The distribution functions, $\left\{f_{M}^{\prime }\left(\vec{v},t\right)\right\}$, depend implicitly on the position of the cells, relative to the global system, and on the velocity $\vec{v}$ and time *t*. Notice that each $f_{M}^{\prime }$ can be formally extended to the complete coordinate space by defining each $f_{M}^{\prime }$ to be zero outside the *M*-th cell, in such a manner that the distribution function of the complete system is a piece-wise sum of $\left\{f_{M}^{\prime }\left(\vec{v},t\right)\right\}$:

(6) $$f^{\prime }\left(\vec{r},\vec{v},t\right)=\sum_{M=1}^{K}f^{\prime }\left({\vec{r}}_{M},\vec{v},t\right)=\sum_{M=1}^{K}f_{M}^{\prime }\left(\vec{v},t\right).$$

Here, ${\vec{r}}_{M}$ is the center of the cell of index *M*, and $f_{M}^{\prime }\left(\vec{v},t\right)\neq f_{N}^{\prime }\left(\vec{v},t\right)$ for M≠N. This extended definition allows us to omit the symbol $\delta V_{M}$ in all integrals performed over the cell volume. In terms of $f_{M}^{\prime }\left(\vec{v},t\right)$ and a local variable of energy, $\epsilon (\vec{v})$, we have

(7) $$\begin{array}{ccc} H_{M}^{\prime } & = & \int f_{M}^{\prime }\left(\vec{v},t\right)\ln f_{M}^{\prime }\left(\vec{v},t\right)d^{3}v, \end{array}$$

(8) $$\begin{array}{ccc} N_{M}^{\prime } & = & \int f_{M}^{\prime }\left(\vec{v},t\right)d^{3}v, \end{array}$$

(9) $$\begin{array}{ccc} E_{M}^{\prime } & = & \int f_{M}^{\prime }\left(\vec{v},t\right)\epsilon \left(\vec{v}\right)d^{3}v. \end{array}$$

Notice that assuming every $f_{M}^{\prime }$ to be homogeneous implies that we are accepting the validity of the local equilibrium hypothesis. In addition, the set $\left\{f_{M}^{\prime }\left(\vec{v},t\right)\right\}$ must satisfy the following restrictions:

(10) $$\sum_{M=1}^{K}\int f_{M}^{\prime }\left(\vec{v},t\right)d^{3}v=N$$

and

(11) $$\sum_{M=1}^{K}\int f_{M}^{\prime }\left(\vec{v},t\right)\epsilon \left(\vec{v}\right)d^{3}v=E.$$

We now use the variational method to find the extremal of $H^{\prime }$, consistent with restrictions (7)–(11) together with the corresponding Lagrange multipliers $\left\{\alpha_{M}\right\}$ and $\left\{\beta_{M}\right\}$. This yields

(12) $$\begin{array}{ccc} \frac{\delta H^{\prime }}{\delta f_{J}^{\prime }\left({\vec{v}}^{\prime }\right)} & = & \sum_{M=1}^{K}\int \frac{\delta }{\delta f_{J}^{\prime }\left({\vec{v}}^{\prime }\right)}\left[f_{M}^{\prime }\left(\vec{v}\right)\ln f_{M}^{\prime }\left(\vec{v}\right)\right]d^{3}v-\sum_{M=1}^{K}\alpha_{M}\int \frac{\delta f_{M}^{\prime }\left(\vec{v}\right)}{\delta f_{J}^{\prime }\left({\vec{v}}^{\prime }\right)}d^{3}v\\ & & -\sum_{M=1}^{K}\beta_{M}\int \epsilon \left(\vec{v}\right)\frac{\delta f_{M}^{\prime }\left(\vec{v}\right)}{\delta f_{J}^{\prime }\left({\vec{v}}^{\prime }\right)}d^{3}v\\ & = & \ln f_{J}^{\prime }\left({\vec{v}}^{\prime }\right)+1-\alpha_{J}-\beta_{J}\epsilon \left({\vec{v}}^{\prime }\right)=0. \end{array}$$

Solving the last line for $f_{J}^{\prime }\left({\vec{v}}^{\prime }\right)$ renders

(13) $$f_{J}^{\prime }\left({\vec{v}}^{\prime }\right)=C\exp\left(\alpha_{J}+\beta_{J}\epsilon \left({\vec{v}}^{\prime }\right)\right)$$

where *C* is a constant. We notice that by applying the variational procedure on H′, we predict that when equilibrium is reached, the distribution function of each cell has the form of the Maxwell–Boltzmann distribution function, which is consistent with the local equilibrium assumption.

#### 2.3.1. Properties of $H^{\prime }$ for Systems in Equilibrium

If the complete system is in equilibrium without external forces applied to the gas, from classical thermodynamics of systems in equilibrium, we ascertain that the local number of particles and the local energy do not depend on the cell number. In a statistical sense, this is

(14) $$N_{M}^{\prime }=N^{\prime }\equiv {\bar{N}}^{\prime }$$

and

(15) $$\;E_{M}^{\prime }=E^{\prime }\equiv {\bar{E}}^{\prime }.$$

In Equations (14) and (15) the bar implies averaged properties over the complete system. Moreover, the global distribution function is homogeneous, hence $f_{M}^{\prime }$ does not depend on the cell number *M* (i.e., $f_{M}^{\prime }\left(\vec{v},t\right)=f^{\prime }\left(\vec{v},t\right)$, ∀M). Several properties arise directly from this, *e.g.,* from Equations (14) and (15) $E=\sum_{M}E_{M}^{\prime }=K{\bar{E}}^{\prime }$, $N=\sum_{M}N_{M}^{\prime }=K{\bar{N}}^{\prime }$. Here we have used Equations (8) and (9). Equation (5), in terms of Equation (3), can be rewritten as:

(16) $$H^{\prime }\left(t\right)=\int \sum_{M=1}^{K}\left[f^{\prime }\left(\vec{v},t\right)\ln f^{\prime }\left(\vec{v},t\right)\right]d^{3}v=K\int f^{\prime }\left(\vec{v},t\right)\ln f^{\prime }\left(\vec{v},t\right)d^{3}v=KH_{B}\left(t\right).$$

To identify $H^{\prime }$ with the entropy, we need to show that $H^{\prime }\left(t\right)$ is extensive, with respect to $Kf^{\prime }\left(\vec{v},t\right)$. This is shown by analyzing the following expression:

(17) $$\begin{array}{ccc} \int \left[Kf^{\prime }\left(\vec{v},t\right)\right]\ln\left[Kf^{\prime }\left(\vec{v},t\right)\right]d^{3}v & = & \int \left[\left(K\ln K\right)f^{\prime }\left(\vec{v},t\right)+Kf^{\prime }\left(\vec{v},t\right)\ln f^{\prime }\left(\vec{v},t\right)\right]d^{3}v\\ & = & K\int \left\{f^{\prime }\left(\vec{v},t\right)\left[\ln K+\ln f^{\prime }\left(\vec{v},t\right)\right]\right\}d^{3}v. \end{array}$$

We observe that if the number of particles in the μ-space, $f^{\prime }\left(\vec{v},t\right)$, is much larger than the number of cells, *K*, then the first term of Equation (17) is negligible, and consequently:

(18) $$\int \left[Kf^{\prime }\left(\vec{v},t\right)\right]\ln\left[Kf^{\prime }\left(\vec{v},t\right)\right]d^{3}v\approx K\int f^{\prime }\left(\vec{v},t\right)\ln f^{\prime }\left(\vec{v},t\right)d^{3}v,$$

i.e., $H_{M}^{\prime }$ is extensive, and the sum $\sum_{M}H_{M}^{\prime }$ is the *H*-functional of the complete system, which reduces to the Boltzmann *H*-functional. Therefore, H′ can be identified with the entropy density of the system.

Furthermore, in equilibrium, the Lagrange multipliers are position- and time-independent, thus $f_{M}^{\prime }\left(\vec{v}\right)$ reduces to

(19) $$f_{M}^{\prime }\left(\vec{v}\right)=C\exp\left(\alpha +\beta \epsilon (\vec{v})\right)\equiv {\bar{f}}^{\prime }\left(\vec{v}\right),\;M=1,\;\cdots \;,K.$$

The constant *C* can be omitted, which is shown by defining the following $H^{\prime \prime }$ functional:

(20) $$H^{\prime \prime }\left(t\right)=\sum_{M=1}^{K}\int \left[f_{M}^{\prime }\left(\vec{v},t\right)\ln f_{M}^{\prime }\left(\vec{v},t\right)-f_{M}^{\prime }\left(\vec{v},t\right)\right]d^{3}v.$$

Since $\sum_{M=1}^{K}\int f_{M}^{\prime }\left(\vec{v},t\right)d^{3}v=N$ (a constant), and because we are mainly interested in the time-derivative of $H^{\prime \prime }$, *C* can be conveniently omitted. In other words, $H^{\prime }$ leads to the Maxwell–Boltzmann distribution function of systems in equilibrium.

#### 2.3.2. Proof of the *H*-Theorem for Non-Homogeneous Distributions

Throughout this section, we consider a classical gas with an initial condition close to the equilibrium, which ensures that the local equilibrium hypothesis remains valid during the time-evolution of the system. Also, we use the following definitions for the deviations of concentration and energy, relative to the equilibrium values:

(21) $$N_{M}^{\prime }\left(t\right)=\int f_{M}^{\prime }\left(\vec{v},t\right)d^{3}v={\bar{N}}^{\prime }+\Delta_{M}^{\prime }\left(t\right)$$

and

(22) $$E_{M}^{\prime }\left(t\right)=\int f_{M}^{\prime }\left(\vec{v},t\right)\epsilon \left(\vec{v}\right)d^{3}v={\bar{E}}^{\prime }+\delta_{M}^{\prime }\left(t\right).$$

Here ${\bar{N}}^{\prime }=N/K$ and ${\bar{E}}^{\prime }=E/K$ are the cell particle number and the cell energy in equilibrium, respectively, which are given by

(23) $${\bar{N}}^{\prime }=\int {\bar{f}}^{\prime }\left(\vec{v}\right)d^{3}v\;\mathrm{and}\;{\bar{E}}^{\prime }=\int {\bar{f}}^{\prime }\left(\vec{v}\right)\epsilon \left(\vec{v}\right)d^{3}v,$$

where we have used ${\bar{f}}^{\prime }\left(\vec{v}\right)$ as defined in Equation (19). In Equations (21) and (22), $\Delta_{M}^{\prime }$ and $\delta_{M}^{\prime }$ are considered deviations relative to ${\bar{N}}^{\prime }$ and ${\bar{E}}^{\prime }$, respectively. For systems that are sufficiently close to equilibrium, it is reasonable to expect first that $\Delta_{M}^{\prime }\left(t\right)\ll {\bar{N}}^{\prime }$ and $\delta_{M}^{\prime }\left(t\right)\ll {\bar{E}}^{\prime }$, and second that $\Delta_{M}^{\prime }$ and $\delta_{M}^{\prime }$ are sufficiently large compared to the fluctuations of ${\bar{N}}^{\prime }$ and ${\bar{E}}^{\prime }$. Similarly, we can assume that every local distribution function can be written as

(24) $$f_{M}^{\prime }\left(\vec{v},t\right)={\bar{f}}^{\prime }\left(\vec{v}\right)\left(1+g_{M}^{\prime }\left(\vec{v},t\right)\right),\;1\gg \left|g_{M}^{\prime }\left(\vec{v},t\right)\right|.$$

With the previous considerations, in the following, we proof an alternative *H*-theorem, considering the *H*-functional, $H^{\prime }$, defined by Equation (5).

We commence by differentiating Equation (5) with respect to time:

(25) $$\frac{dH^{\prime }}{dt}=\sum_{M=1}^{K}\int \left[1+\ln f_{M}^{\prime }\left(\vec{v},t\right)\right]{\dot{f}}_{M}^{\prime }\left(\vec{v},t\right)d^{3}v.$$

(Starting here, we use the standard notation $\dot{h}\equiv \left(dh/dt\right)$). Substituting Equation (24) into Equation (25) yields

(26) $$\frac{dH^{\prime }}{dt}=\sum_{M=1}^{K}\int {\bar{f}}^{\prime }\left(\vec{v}\right)\left[1+\ln\left\{{\bar{f}}^{\prime }\left(\vec{v}\right)+{\bar{f}}^{\prime }\left(\vec{v}\right)g_{M}^{\prime }\left(\vec{v},t\right)\right\}\right]{\dot{g}}_{M}^{\prime }\left(\vec{v},t\right)d^{3}v.$$

The logarithmic term of Equation (26) expanded up to the first-order term of its Taylor series, around $g_{M}^{\prime }\left(\vec{v},t\right)=0$, is

(27) $$\ln\left[{\bar{f}}^{\prime }\left(\vec{v}\right)+{\bar{f}}^{\prime }\left(\vec{v}\right)g_{M}^{\prime }\left(\vec{v},t\right)\right]\approx \ln\left[{\bar{f}}^{\prime }\left(\vec{v}\right)\right]+g_{M}^{\prime }\left(\vec{v},t\right),$$

and substituting this into Equation (26) gives

(28) $$\begin{array}{ccc} \frac{dH^{\prime }}{dt} & = & \sum_{M=1}^{K}\int {\bar{f}}^{\prime }\left(\vec{v}\right)\left[1+\ln{\bar{f}}^{\prime }\left(\vec{v}\right)+g_{M}^{\prime }\left(\vec{v},t\right)\right]{\dot{g}}_{M}^{\prime }\left(\vec{v},t\right)d^{3}v. \end{array}$$

Substituting $\ln{\bar{f}}^{\prime }\left(\vec{v}\right)=\exp\left(\alpha +\beta \epsilon (\vec{v})\right)$, see Equation (19) and subsequent text, and omitting *C* we obtain

(29) $$\frac{dH^{\prime }}{dt}=\sum_{M=1}^{K}\int \bar{f}\left(\vec{v}\right)\left[\alpha +\beta \epsilon (\vec{v})\right]{\dot{g}}_{M}\left(\vec{v},t\right)d^{3}v+\sum_{M=1}^{K}\int \bar{f}\left(\vec{v}\right)g_{M}\left(\vec{v},t\right){\dot{g}}_{M}\left(\vec{v},t\right)d^{3}v.$$

From the definitions of $N_{M}^{\prime }$ and $E_{M}^{\prime }$ —Equations (21) and (22)—and $f_{M}^{\prime }\left(\vec{v},t\right)$ —Equation (24)—it is straightforward to show that

(30) $$\begin{array}{ccc} \int \bar{f}\left(\vec{v}\right)g_{M}\left(\vec{v},t\right)d^{3}v=\Delta_{M}\left(t\right)\;\; & \Rightarrow & \;\;\int \bar{f}\left(\vec{v}\right){\dot{g}}_{M}\left(\vec{v},t\right)d^{3}v={\dot{\Delta }}_{M}\left(t\right), \end{array}$$

(31) $$\begin{array}{ccc} \int \bar{f}\left(\vec{v}\right)g_{M}\left(\vec{v},t\right)\epsilon \left(\vec{v}\right)d^{3}v=\delta_{M}\left(t\right)\;\; & \Rightarrow & \;\;\int \bar{f}\left(\vec{v}\right){\dot{g}}_{M}\left(\vec{v},t\right)\epsilon \left(\vec{v}\right)d^{3}v={\dot{\delta }}_{M}\left(t\right), \end{array}$$

and as a consequence of $\sum_{M=1}^{K}\Delta_{M}\left(t\right)=\sum_{M=1}^{K}\delta_{M}\left(t\right)=0$, we find

(32) $$\sum_{M=1}^{K}{\dot{\Delta }}_{M}\left(t\right)=\sum_{M=1}^{K}{\dot{\delta }}_{M}\left(t\right)=0.$$

Therefore, due to Equations (30)–(32), Equation (29) simplifies to

(33) $$\frac{dH^{\prime }}{dt}=\sum_{M=1}^{K}\int \bar{f}\left(\vec{v}\right)g_{M}\left(\vec{v},t\right){\dot{g}}_{M}\left(\vec{v},t\right)d^{3}v.$$

To clearly determine the time-evolution of Equation (33), we split the summation over *M* into two terms:

(34) dH′dt=∑JL∫f¯′(v→)gI′+(v→,t)g˙′I.+(v→,t)d3v+∑JP∫f¯′(v→)gJ′−(v→,t)g˙′J.−(v→,t)d3v

where L+P=K. The above split is made based on the assumption that for any given initial state of the system, at $t_{0}$, some cells will have either a $g_{I}^{\prime }\left(\vec{v},t_{0}\right)\geq 0$ or a $g_{J}^{\prime }\left(\vec{v},t_{0}\right)<0$, which we denote as g˙I′.+(v→,t) or g˙J′.−(v→,t), respectively.

If the system’s initial state is sufficiently close to equilibrium, it is physically appropriate to assume that $\left|g_{M}^{\prime }\left(\vec{v},t_{0}\right)\right|\to 0$ as t→∞ in a monotonous manner, thus g˙I′.+(v→,t)≤0 and g˙J′.−(v→,t)>0, for $t\geq t_{0}$. Consequently, Equation (34) can be re-written as

(35) $$\frac{dH^{\prime }}{dt}=-\left[\sum_{J}^{L}\int \bar{f}\left(\vec{v}\right)|g_{J}^{+}\left(\vec{v},t\right)\left|\right|{\dot{g}}_{J}^{+}\left(\vec{v},t\right)|d^{3}v+\sum_{J}^{P}\int \bar{f}\left(\vec{v}\right)|g_{J}^{-}\left(\vec{v},t\right)\left|\right|{\dot{g}}_{J}^{-}\left(\vec{v},t\right)|d^{3}v\right].$$

Since every integrand in Equation (35) is positive, for all *t* and $\vec{v}$, and $\left|g_{M}^{\prime }\left(\vec{v},t_{0}\right)\right|\to 0$ as t→∞, it follows that

(36) $$\frac{dH^{\prime }}{dt}\leq 0.\;\;\mathrm{QED}.$$

In summary, considering a gas occupying a volume V (which is divided into *K* small cells), with a total energy *E* and *N* classical free particles, whose initial state is not in equilibrium, but sufficiently close to equilibrium, then the functional

(37) $$H^{\prime }\left(t\right)=\sum_{M=1}^{K}\int f_{M}^{\prime }\left(\vec{v},t\right)\ln f_{M}^{\prime }\left(\vec{v},t\right)d^{3}v$$

where $f_{M}^{\prime }\left(\vec{v},t\right)$ is the cell distribution function, which satisfies $dH^{\prime }/dt\leq 0$, and the equality relation is attained at t→∞. In Equation (37), $f_{M}^{\prime }\left(\vec{v},t\right)$ is the Maxwell–Boltzmann distribution function, which in general is different for different cells—i.e., the complete system can be non-homogeneous—and each $f_{M}^{\prime }\left(\vec{v},t\right)$ is compatible with the cell properties, such as number of particles, $N_{M}^{\prime }$, energy, $E_{M}^{\prime }$, temperature, $T_{M}$, and Legendre multipliers $\alpha_{M}$ and $\beta_{M}$.

## 3. Quantum Scheme

The classical *H*-theorem is still considered one pillar on which classical statistical physics is founded. Unfortunately, despite multiple attempts [3,10,11,14,15,16], the generality of the classical *H*-theorem has no equally robust quantum match. In this section, we propose and analyze an alternative quantum *H*-functional using the variational method. We start by briefly outlining a typical textbook demonstration of the quantum *H*-theorem [2], and subsequently present the analysis of our proposed *H*-functional.

### 3.1. *H*-Theorem and the Fermi–Dirac and Bose–Einstein Distribution Functions

Consider a dilute gas of *N* non-interacting quantum particles (either bosons or fermions), contained by a vessel of volume *V*, temperature *T*, and total energy E. Starting from the Boltzmann definition of entropy, the quantum *H* functional is

(38) $$H_{T}=-\ln G,$$

where *G* describes the total number of accessible quantum states of the gas that satisfy the above conditions [2]. The quantum *H*-theorem can be demonstrated as follows. *G* can be divided into groups of neighboring states, $g_{k}$, and certain occupation numbers, $n_{k}$, can be associated with each of these groups. Thus, the above functional takes the form

(39) $$H_{T}=\sum_{i}n_{i}\ln n_{i}-\left(n_{i}\pm g_{i}\right)\ln\left(g_{i}\pm n_{i}\right)\pm g_{i}\ln g_{i},$$

where the upper and lower signs are for bosons and fermions, respectively. Thus the time derivative of Equation (39) is

(40) $$\frac{dH_{T}}{dt}=\sum_{\kappa }\left[\ln n_{\kappa }-\ln\left(g_{\kappa }\pm n_{\kappa }\right)\right]\frac{dn_{\kappa }}{dt}.$$

Assuming that the energy exchange between particles is produced by interparticle collisions, and using perturbation theory, the rate of change in the number of particles in a group κ is

(41) $$\begin{array}{ccc} \frac{dn_{\kappa }}{dt} & = & -\sum_{\lambda ,(\mu \nu )}A_{\kappa \lambda ,\mu \nu }n_{\kappa }n_{\lambda }\left(g_{\mu }\pm n_{\mu }\right)\left(g_{\nu }\pm n_{\nu }\right)\\ & & +\sum_{\lambda ,(\mu \nu )}A_{\mu \nu ,\kappa \lambda }n_{\mu }n_{\nu }\left(g_{\kappa }\pm n_{\kappa }\right)\left(g_{\lambda }\pm n_{\lambda }\right). \end{array}$$

Here $A_{\kappa \lambda ,\mu \nu }n_{\kappa }n_{\lambda }\left(g_{\mu }\pm n_{\mu }\right)\left(g_{\nu }\pm n_{\nu }\right)$ is the expected number of collisions per unit time, in which two particles will be moved from groups (κ, λ) to (μ, ν), and the tensor $A_{\kappa \lambda ,\mu \nu }$ is given by

(42) $$A_{\kappa \lambda ,\mu \nu }=\frac{4\pi^{2}}{h}\frac{|I_{1}\pm I_{2}{|}^{2}}{\Delta \epsilon }.$$

In Equation (42), Δϵ is the net energy change occurring during the collision and $|I_{1}-I_{2}{|}^{2}={\left|V_{mn,kl}\right|}^{2}$, where $V_{mn,kl}$ is the element of the transition matrix of a binary collision. It is important to remark that in deriving Equation (41), the equal a priori probabilities and the random a priori phase hypotheses were assumed valid. The random a priori phase hypothesis can be considered an analogue of the molecular chaos hypothesis [15], as it is the mechanism by which stochasticity is introduced into the system.

Substituting Equation (41) into Equation (40), it is straightforward to prove that

(43) $$\frac{dH_{T}}{dt}\leq 0.$$

At equilibrium (at t→∞), $dn_{\kappa }/dt=0$, hence from Equation (41)

(44) $$\ln\frac{n_{\kappa }}{g_{\kappa }\pm n_{\kappa }}+\ln\frac{n_{\lambda }}{g_{\lambda }\pm n_{\lambda }}=\ln\frac{n_{\mu }}{g_{\mu }\pm n_{\nu }}+\ln\frac{n_{\nu }}{g_{\nu }\pm n_{\nu }}.$$

Considering that energy is conserved during the collision, the Bose–Einstein or Fermi–Dirac distribution functions can be recovered from Equation (44):

(45) $$n_{\kappa }=\frac{g_{\kappa }}{\exp(\alpha +\beta \epsilon_{\kappa })\mp 1}.$$

In other words, at equilibrium $dH_{T}/dt=0$, the distribution function obtained from Equation (39) is the expected distribution function.

### 3.2. Out-of-Equilibrium, Non-Homogeneous Quantum Systems

Consider a dilute gas enclosed by a perfectly isolated vessel of volume *V*, with total energy *E*, and total number of quantum particles *N*, which can be free fermions or bosons. For our purposes, the volume *V* is divided into *K* small cells, each of which has constant volume $\delta V_{M}=V/K$ (M=1,⋯,K), temperature $T_{M}$, energy $\epsilon_{M}$, number of particles $N_{M}$, and distribution function, $\left\{f_{Mn}\left(t\right)\right\}$. Hereafter we use the following short-hand notation:

(46) $$f_{Mn}\left(t\right)\equiv f\left({\vec{r}}_{M},\epsilon_{n},t\right),$$

where ${\vec{r}}_{M}$ is the radius vector pointing at the center of the *M*-th cell. $f_{Mn}\left(t\right)$ represents the number of particles contained in the *M*-th cell that occupies the energy level $\epsilon_{n}$ at time *t*. Since the particles are considered to be free, the energy levels should not depend on the cell properties, i.e., the energy spectrum, $\left\{\epsilon_{n}\right\}$, is the same for all cells; thus, there is no need to label $\epsilon_{n}$ with an index *M*.

We propose the following functional as an alternative *H*-functional for quantum non-homogeneous dilute gases:

(47) $$\begin{array}{ccc} H(t) & = & \sum_{M=1}^{K}\sum_{n}[f_{Mn}\left(t\right)\ln f_{Mn}\left(t\right)\\ & & \;\;\pm \left(1\mp f_{Mn}\left(t\right))\ln(1\mp f_{Mn}\left(t\right)\right)]\delta V_{M}. \end{array}$$

Here, the upper and lower signs refer to fermions and bosons, respectively. In addition, when needed, each cell has an associated local chemical potential, $\alpha_{M}$, and a local *H*-functional, which is defined by

(48) $$H_{M}\left(t\right)=\sum_{n}\left[f_{Mn}\left(t\right)\ln f_{Mn}\left(t\right)\pm \left(1\mp f_{Mn}\left(t\right)\right)\ln\left(1\mp f_{Mn}\left(t\right)\right)\right]\delta V_{M}.$$

Therefore, $N_{M}$ and $E_{M}$ as functions of time are given by

(49) $$N_{M}\left(t\right)=\sum_{n}f_{Mn}\left(t\right)\delta V_{M}$$

and

(50) $$E_{M}\left(t\right)=\sum_{n}f_{Mn}\left(t\right)\epsilon_{n}\delta V_{M},$$

which are, for the whole system, constrained by the micro-canonical restrictions

(51) $$\sum_{M=1}^{K}\left[\sum_{n}f_{Mn}\left(t\right)\right]\delta V_{M}=\sum_{M=1}^{K}N_{M}\left(t\right)\delta V_{M}=N,$$

and

(52) $$\sum_{M=1}^{K}\left[\sum_{n}f_{Mn}\left(t\right)\epsilon_{n}\right]\delta V_{M}=\sum_{M=1}^{K}E_{M}\left(t\right)\delta V_{M}=E.$$

Applying the variational method to H, and using the Lagrange multipliers $\left\{\alpha_{M}\right\}$ and $\left\{\beta_{M}\right\}$, we readily obtain (see also the discussion related to Equation (12)):

(53) $$\ln\left(\frac{1\mp f_{Mn}\left(t\right)}{f_{Mn}\left(t\right)}\right)=-\alpha_{M}\left(t\right)-\beta_{M}\left(t\right)\epsilon_{n},$$

and solving for $f_{Mn}\left(t\right)$ yields

(54) $$f_{Mn}\left(t\right)=\frac{1}{\exp\left(-\alpha_{M}\left(t\right)-\beta_{M}\left(t\right)\epsilon_{n}\right)\pm 1}.$$

Thus, in this zero-order approximation, the form of equilibrium distribution functions is conserved.

#### 3.2.1. Properties of H for Systems in Equilibrium

If the system is in equilibrium, the temperature becomes homogeneous throughout the complete system. Also, the local number of particles, the local energy, and the Lagrange multipliers do not depend on the cell number, and they should be homogeneous. This is represented by

(55) $$N_{M}\left(t\to \infty \right)\equiv \bar{N}=N/K,$$

(56) $$E_{M}\left(t\to \infty \right)\equiv \bar{E}=E/K,$$

(57) $$\alpha_{M}=\bar{\alpha },\;\forall M,$$

and

(58) $$\beta_{M}=\bar{\beta },\;\forall M.$$

Substituting Equations (57) and (58) into Equation (54) yields the distribution function of each cell in equilibrium:

(59) $${\bar{f}}_{Mn}={\bar{f}}_{n}=\frac{1}{\exp\left(-\bar{\alpha }-\bar{\beta }\epsilon_{n}\right)\pm 1},\;\forall M.$$

Using the above equation, we can recover the distribution function and the entropy of a dilute quantum gas in equilibrium as follows. Setting $\bar{\alpha }=\mu /kT$ and $\bar{\beta }=-1/kT$, and substituting them into Equation (59), it renders the Fermi–Dirac and Bose–Einstein distribution functions:

(60) $${\bar{f}}_{n}=\frac{1}{\exp\left(\frac{\epsilon_{n}-\mu }{kT}\right)\pm 1},$$

and substituting Equation (59) into the negative of Equation (47), the entropy of a quantum ideal gas is

(61) $$\begin{array}{ccc} S & = & \sum_{M=1}^{K}\sum_{n}\left[\left(\frac{1}{\exp\left(-\bar{\alpha }-\bar{\beta }\epsilon_{n}\right)\pm 1}\right)\ln\left(\frac{1}{\exp\left(-\bar{\alpha }-\bar{\beta }\epsilon_{n}\right)\pm 1}\right)\right]\\ & & \;\pm \ln\left[\prod_{M=1}^{K}\prod_{n}\left(1\mp \frac{1}{\exp\left(-\bar{\alpha }-\bar{\beta }\epsilon_{n}\right)\pm 1}\right)\right]\delta V_{M}. \end{array}$$

This quantity is what we refer to as “variational entropy,” and this name reflects the fact that it was obtained via the variational method.

#### 3.2.2. Proof of the Quantum *H*-Theorem for Non-Homogeneous Systems

For quantum systems, we also accept the validity of the local equilibrium hypothesis for every cell in the system. This allows us to define non-homogeneous systems, wherein thermodynamic quantities are well-defined on a per-cell basis. In terms of the equilibrium properties, we have

(62) $$N_{M}\left(t\right)=\sum_{n}f_{Mn}\left(t\right)=\bar{N}+\Delta_{M}\left(t\right)$$

and

(63) $$E_{M}\left(t\right)=\sum_{n}\epsilon_{n}f_{Mn}\left(t\right)=\bar{E}+\delta_{M}\left(t\right).$$

In Equations (62) and (63) $\bar{N}$ and $\bar{E}$ are the cell particle number and the cell energy in equilibrium, which are given by Equations (55) and (56), and $\Delta_{M}$ and $\delta_{M}$ are deviations from $\bar{N}$ and $\bar{E}$, respectively, with $\Delta_{M}\left(t\right)\ll \bar{N}$ and $\delta_{M}\left(t\right)\ll \bar{E}$.

In the present context, $|\Delta_{M}|$ and $|\delta_{M}|$ are sufficiently large to not be fluctuations of the system, and sufficiently small so that the local equilibrium hypothesis is valid for t>0 (we set $t_{0}=0$, and $t_{0}$ is the initial time at which the system is prepared). Therefore, the distribution functions can be rewritten as

(64) $$f_{Mn}\left(t\right)={\bar{f}}_{n}\left(1+g_{Mn}\left(t\right)\right),\;1\gg \left|g_{Mn}\left(t\right)\right|,$$

from which it follows, by substituting Equation (64) into Equations (62) and (63), that $\Delta_{M}$ and $\delta_{M}$ satisfy

(65) $$\Delta_{M}\left(t\right)=\sum_{n}{\bar{f}}_{n}g_{nM}$$

and

(66) $$\delta_{M}\left(t\right)=\sum_{n}{\bar{f}}_{n}g_{nM}\epsilon_{n}.$$

An additional consideration is necessary for treating Fermi gases. Since, for these systems, $f_{Mn}\left(t\right)\leq 1$, we have

(67) $$\begin{array}{c} 1-{\bar{f}}_{n}-{\bar{f}}_{n}g_{nM}\geq 0\;\;\Rightarrow \;\;\frac{1}{{\bar{f}}_{n}}\geq 1+g_{nM}. \end{array}$$

${\bar{f}}_{n}=1$ is certainly satisfied if the system temperature is zero. In this state, all energy levels below and including the Fermi energy are occupied, thus the system will necessarily be homogeneous, and consequently, $g_{nM}=0$. In this article, we will omit this scenario and will only discuss Fermi gases with non-zero temperatures.

To proof the quantum *H*-theorem, we start by taking the time-derivative of Equation (47):

(68) $$\frac{dH(t)}{dt}=\sum_{n}\sum_{M=1}^{K}{\dot{f}}_{nM}\left(t\right)\ln\left[\frac{f_{nM}\left(t\right)}{1\mp f_{nM}\left(t\right)}\right]\delta V_{M}.$$

Subsequently, we substitute Equation (64) into the above equation to obtain

(69) $$\begin{array}{ccc} \frac{dH(t)}{dt} & = & \sum_{n}\sum_{M=1}^{K}{\bar{f}}_{n}\ln\left[\frac{{\bar{f}}_{n}\left(1+g_{nM}\right)}{1\mp {\bar{f}}_{n}\left(1+g_{nM}\right)}\right]{\dot{g}}_{nM}\delta V_{M}\\ & = & \sum_{n}\sum_{M=1}^{K}{\bar{f}}_{n}\left\{\ln\left[{\bar{f}}_{n}+{\bar{f}}_{n}g_{nM}\right]{\dot{g}}_{nM}-\ln\left[1\mp {\bar{f}}_{n}\mp {\bar{f}}_{n}g_{nM}\right]{\dot{g}}_{nM}\right\}\delta V_{M}. \end{array}$$

The logarithmic terms, corresponding to Fermi and Bose gases, are approximated through a Taylor series around ${\bar{f}}_{n}g_{nM}=0$ as

(70) $$\ln\left[1\mp {\bar{f}}_{n}\mp {\bar{f}}_{n}g_{nM}\right]\approx \ln\left[1\mp {\bar{f}}_{n}\right]\mp \frac{{\bar{f}}_{n}}{1\mp {\bar{f}}_{n}}g_{nM}$$

and

(71) $$\ln\left[{\bar{f}}_{n}+{\bar{f}}_{n}g_{nM}\right]\approx \ln\left[{\bar{f}}_{n}\right]+g_{nM},$$

respectively. Equation (70) is valid because, for non-extremely degenerated Fermi gases, $1-{\bar{f}}_{n}\gg {\bar{f}}_{n}\left|g_{nM}\right|$ and Equation (71) is fulfilled because, for Boson gases, $1+{\bar{f}}_{n}\gg {\bar{f}}_{n}\left|g_{nM}\right|$ when ${\bar{f}}_{n}\gg {\bar{f}}_{n}\left|g_{nM}\right|$.

Combining Equations (69)–(71),

(72) $$\begin{array}{ccc} \frac{dH}{dt} & = & \sum_{n}\sum_{M=1}^{K}{\bar{f}}_{n}\left\{\left(\ln{\bar{f}}_{n}+g_{nM}\right){\dot{g}}_{nM}\right\}\delta V_{M}\delta \epsilon_{n}\\ & & -\sum_{n}\sum_{M=1}^{K}{\bar{f}}_{n}\left\{\left(\ln\left[1\mp {\bar{f}}_{n}\right]\mp \left[\frac{{\bar{f}}_{n}}{1\mp {\bar{f}}_{n}}\right]g_{nM}\right){\dot{g}}_{nM}\right\}\delta V_{M} \end{array}$$

and substituting Equation (59) into Equation (72):

(73) $$\frac{dH}{dt}=\sum_{n}\sum_{M=1}^{K}{\bar{f}}_{n}\left\{\left(\bar{\alpha }+\bar{\beta }\epsilon_{n}\right){\dot{g}}_{nM}+g_{nM}\left(1\pm e^{\bar{\alpha }+\bar{\beta }\epsilon_{n}}\right){\dot{g}}_{nM}\right\}\delta V_{M}.$$

Since both the total number of particles and the total energy of the system are constant, it follows from Equations (51), (52), (62) and (63) that

(74) $$\frac{dN}{dt}=\sum_{M=1}^{K}{\dot{N}}_{M}\delta V_{M}=\sum_{M=1}^{K}\sum_{n}{\bar{f}}_{n}{\dot{g}}_{nM}\delta V_{M}=\sum_{M=1}^{K}{\dot{\Delta }}_{M}\left(t\right)\delta V_{M}=0$$

and

(75) $$\frac{dE}{dt}=\sum_{M=1}^{K}{\dot{E}}_{M}\delta V_{M}=\sum_{M=1}^{K}\sum_{n}{\bar{f}}_{n}{\dot{g}}_{nM}\epsilon_{n}\delta V_{M}=\sum_{M=1}^{K}{\dot{\delta }}_{M}\left(t\right)\delta V_{M}=0.$$

Substitute the previous expression in Equation (73) to obtain

(76) $$\frac{dH}{dt}=\sum_{n}e^{\bar{\alpha }+\bar{\beta }\epsilon_{n}}\sum_{M=1}^{K}g_{nM}{\dot{g}}_{nM}\delta V_{M}\leq 0.\;\;\mathrm{QED}.$$

To obtain the far right side of Equation (76), we have used the relationship $g_{nM}{\dot{g}}_{nM}\leq 0$ for t>0. This can be proven by simply arguing that, in the initial state, if a cell is described by $g_{nM}\left(t_{0}\right)>0$ then $g_{nM}\left(t\right)\geq 0$ and ${\dot{g}}_{nM}\left(t\right)\leq 0$, and if $g_{nM}\left(t_{0}\right)<0$ then $g_{nM}\left(t\right)\leq 0$ and ${\dot{g}}_{nM}\left(t\right)\geq 0$. Here we have exploited the fact that the system in equilibrium is homogeneous, and that, by accepting the local equilibrium hypothesis, $g_{Mn}\left(t\right)$ is a monotonic function and $g_{nM}\to 0$ as t→∞ as the system approaches the equilibrium state. Another approach to prove Equation (76) consists of splitting the cells into two subsets, just as we did in the classical scenario.

Briefly, considering a dilute quantum gas contained in a vessel of volume *V* (divided into *K* small cells), with total energy *E* and *N* quantum free particles, which initially is out of equilibrium—but in such a manner that the local equilibrium hypothesis is valid— the functional

(77) $$\begin{array}{ccc} H(t) & = & \sum_{M=1}^{K}\sum_{n}\left[f_{Mn}\left(t\right)\ln f_{Mn}\left(t\right)\pm \left(1\mp f_{Mn}\left(t\right))\ln(1\mp f_{Mn}\left(t\right)\right)\right]\delta V_{M}, \end{array}$$

where $f_{Mn}$ is the *M*-th cell distribution function, evolves in time such that dH/dt≤0, and the equality condition is attained when the system reaches the equilibrium state. In Equation (77), and for a Fermi (Bose) gas, $f_{Mn}$ corresponds to the Fermi–Dirac (Bose–Einstein) distribution function for each cell. Locally, each cell is in equilibrium, although the complete system may be non-homogeneous, and is characterized by the respective $f_{Mn}$, number of particles $N_{M}$, energy $E_{M}$, temperature $T_{M}$, and Legendre multipliers $\alpha_{M}$ and $\beta_{M}$.

## 4. Quantum—Classical Correspondence

In Section 2 and Section 3, we saw that the variational method can be applied to *H*-functionals, which correctly describes the behavior of classical and quantum dilute gases, with regard to their respective time-evolution. Both *H*-functionals defined in Equations (5) and (47) also recover the well-known distribution functions, either Maxwell–Boltzmann for a classical gas, Fermi–Dirac for a Fermi gas, or Bose–Einstein for a Bose gas. Nevertheless, the functionals (5) and (47) are seemingly different, and in this section, we show they are related by the correspondence principle.

We start by arguing that, in equilibrium, it is straightforward to proof that Equation (60) collapses into Equation (19) by taking the limit wherein the degeneration parameter $\xi \equiv \exp\left(-\left(\epsilon -\mu \right)/\left(k_{B}T\right)\right)\gg 1$. Alternatively, a more general approach to show the quantum–classical correspondence consists of analyzing the collapse from the quantum to the classical *H*-functionals within the appropriated limit. For the case treated here, this limit is $f_{nM}\approx 0$ for several reasons. Systems at very low temperatures, in which the quantum effects cannot be ignored, are obviously excluded from the current analysis. In systems at sufficiently high temperatures, the particles occupy almost exclusively high-energy levels. Furthermore, the energy spectrum approaches a continuum, as is expected by taking the limit ℏ→0, and the number of particles per level is very close to zero.

Subsequently, we substitute $f_{nM}\approx 0$ into Equation (47) to obtain

(78) $$H=\sum_{M=1}^{K}\sum_{n}\left[f_{nM}\left(t\right)\ln\left(f_{nM}\left(t\right)\right)\right]\delta V_{M}=\sum_{M=1}^{K}\sum_{n}\left[f_{M}\left(\epsilon_{n},t\right)\ln\left(f_{M}\left(\epsilon_{n},t\right)\right)\right]\delta V_{M}.$$

Finally, the sum over the quantum energy levels can be replaced by an integral over the velocities by invoking both the uncertainty principle and the fact that, for free particles, the continuum energy spectrum can be written as a function of the velocity. Hence the quantum *H*-functional transforms, in the classical limit, to

(79) $$H=\sum_{M=1}^{K}\int C^{\prime }f_{M}\left(\vec{v},t\right)\ln\left[C^{\prime }f_{M}\left(\vec{v},t\right)\right]d\vec{v},$$

where $C^{\prime }$ collects the appropriate constants stemming from writing the energy spectrum as a function of $\vec{v}$.

## 5. Relaxation Processes in Degenerated Quantum Gases

To obtain a time-evolution equation for an out-of-equilibrium quantum gas, we propose the following approach. We start by evaluating $\Delta H=H\left(t_{2}\right)-H\left(t_{1}\right)$, where our quantum *H*-functional—Equation (47)—is evaluated at different times $t_{1}$ and $t_{2}$, with $t_{2}>t_{1}$. This yields

(80) $$\begin{array}{ccc} \Delta H & = & \sum_{M=1}^{K}\sum_{n}[f_{nM}^{\prime \prime }\ln f_{nM}^{\prime \prime }-f_{nM}^{\prime }\ln f_{nM}^{\prime }\\ & & \pm \left(1\mp f_{nM}^{\prime \prime }\right)\ln\left(1\mp f_{nM}^{\prime \prime }\right)\mp \left(1\mp f_{nM}^{\prime }\right)\ln\left(1\mp f_{nM}^{\prime }\right)]\delta V_{M}. \end{array}$$

In the above equation, and for the rest of this section, we use the short-hand notation $f_{nM}\left(t_{2}\right)\equiv f_{nM}^{\prime \prime }$ and $f_{nM}\left(t_{1}\right)\equiv f_{nM}^{\prime }$. Subsequently, in Equation (80), we replace the distribution functions with their expressions in terms of deviations from equilibrium—Equation (64)—which renders:

(81) $$\begin{array}{ccc} \Delta H & = & \sum_{M=1}^{K}\sum_{n}[{\bar{f}}_{n}\left(1+g_{nM}^{\prime \prime }\right)\ln{\bar{f}}_{n}\left(1+g_{nM}^{\prime \prime }\right)-{\bar{f}}_{n}\left(1+g_{nM}^{\prime }\right)\ln{\bar{f}}_{n}\left(1+g_{nM}^{\prime }\right)\\ & & \pm \left(1\mp {\bar{f}}_{n}\left\{1+g_{nM}^{\prime \prime }\right\}\right)\ln\left(1\mp {\bar{f}}_{n}\left\{1+g_{nM}^{\prime \prime }\right\}\right)\\ & & \mp \left(1\mp {\bar{f}}_{n}\left\{1+g_{nM}^{\prime }\right\}\right)\ln\left(1\mp {\bar{f}}_{n}\left\{1+g_{nM}^{\prime }\right\}\right).]\delta V_{M}. \end{array}$$

Subsequently, we expand the logarithmic terms up to the first-order in $g_{nM}^{\prime }$ and $g_{nM}^{\prime \prime }$ and rearrange the result, which gives

(82) $$\Delta H=\sum_{M=1}^{K}\sum_{n}\left[{\bar{f}}_{n}\left(1+\ln{\bar{f}}_{n}\right)-{\bar{f}}_{n}\left\{\ln\left(1\mp {\bar{f}}_{n}\right)\mp 1\right\}\right]\left(g_{nM}^{\prime \prime }-g_{nM}^{\prime }\right)\delta V_{M}.$$

Finally we divide Equation (82) by $\Delta t\equiv t_{2}-t_{1}$, and take the limit Δt→0 to obtain

(83) $$\frac{dH}{dt}=\sum_{M=1}^{K}\sum_{n}\left[{\bar{f}}_{n}\left(1+\ln{\bar{f}}_{n}\right)-{\bar{f}}_{n}\left\{\ln\left(1\mp {\bar{f}}_{n}\right)\mp 1\right\}\right]\left(\frac{g_{nM}}{dt}\right)\delta V_{M}.$$

Equation (83) is, within our framework, the time-evolution equation for $g_{nM}$. Clearly, to describe a realistic situation, providing a specific approximation for the deviation function $g_{nM}$ is required. This subject will be explored in future work.

## 6. Comments and Remarks

The demonstration of the classical *H*-theorem usually begins by assuming that the gas, despite being initially out of equilibrium, can be described by a spatially homogeneous distribution function. Subsequently, the time-evolution of the system occurs in such a manner that dH/dt≤0. Therefore, this approach does not describe the evolution to equilibrium of systems with spatial inhomogeneities. To address this issue, in this article, we proposed a framework that may be useful to describe the time-evolution of initially non-homogeneous systems. To this end, we divided the system into small cells to conceive a system wherein the local equilibrium hypothesis is valid in each cell but in such a manner that the total system is not homogeneous. Systems that satisfy the previous conditions will evolve towards equilibrium, and the evolution occurs according to $dH^{\prime }/dt\leq 0$, Equation (5) and dH/dt≤0, and Equation (47), for classical and quantum gases, respectively. Consequently, this approach can be considered an extension of the *H*-theorem for more realistic out-of-equilibrium systems.

The classical and quantum *H*-functionals, $H^{\prime }$ and H, respectively, correctly recover the most-probable distribution functions in out-of-equilibrium states (locally) and when the system attains the global equilibrium state. The relaxation process of the system is described by monotonic functions that account for deviations from the global equilibrium.

It is clear that for describing the relaxation process of a concrete system, it is necessary to know, at least to some approximation, the specific forms of the monotonic functions $g_{M}^{\prime }$ and $g_{nM}$, for classical and quantum systems. Whereas the complete analysis of these functions is beyond the scope of the present work, some of their properties can be predicted, *e.g.,* they must be consistent both with the system relaxation times and the mechanisms of energy transfer between cells.

An important aspect of the framework proposed in this work is related to the entropy of systems out-of-equilibrium. Because the functionals $H^{\prime }$ and H can be related to the entropy of dilute gases, either classical or quantum, the fact that these functionals are defined over a system divided into cells enables their use for defining the entropy of out-of-equilibrium systems, other than dilute gases. Specifically, and derived from our previous work (e.g., [30,31]), the $H^{\prime }$ and H functionals may serve to describe the entropy, as well as the entropy generation, occurring during the growth of complex physical systems, such as fractals. Possibly, studying these systems might also shed light on the explicit functional form of $g_{M}^{\prime }$ and $g_{nM}$.

In summary, we proposed a variational procedure to demonstrate the classical and quantum *H*-theorems, which allowed us to describe, at a mesoscopic local view (cell-scale), the time-evolution of an out-of-equilibrium and spatially non-homogeneous system moving towards the equilibrium condition. In principle, this approach would permit the investigation of the transport phenomena inherent to the equilibration process, occurring in a system with a spatially inhomogeneous out-of-equilibrium initial condition.

## Abbreviations

The following abbreviations are used in this manuscript:

$H_{B}$	The original Boltzmann *H*-functional
$f_{B}=f\left(\vec{v}\right)$	The Maxwell–Boltzmann distribution function
$H^{\prime }$	Our *H*-functional for a classical dilute gas
$f_{M}^{\prime }=f^{\prime }\left({\vec{r}}_{M},\vec{v},t\right)$	The classical distribution function of a cell centered at ${\vec{r}}_{M}$
$H_{T}$	The *H*-functional proposed by Tolman
H	Our *H*-functional for a quantum dilute gas
$f_{nM}=f_{M}\left({\vec{r}}_{M},\epsilon_{n},t\right)$	The quantum distribution function of a cell centered at ${\vec{r}}_{M}$
